# The Gait Disorder in Downbeat Nystagmus Syndrome

**DOI:** 10.1371/journal.pone.0105463

**Published:** 2014-08-20

**Authors:** Roman Schniepp, Max Wuehr, Sabrina Huth, Cauchy Pradhan, Cornelia Schlick, Thomas Brandt, Klaus Jahn

**Affiliations:** 1 Department of Neurology, University of Munich, Munich, Germany; 2 German Center for Vertigo and Balance Disorders (DSGZ), University of Munich, Munich, Germany; 3 Institute for Clinical Neurosciences, University of Munich, Munich, Germany; University of Iowa, United States of America

## Abstract

**Background:**

Downbeat nystagmus (DBN) is a common form of acquired fixation nystagmus with key symptoms of oscillopsia and gait disturbance. Gait disturbance could be a result of impaired visual feedback due to the involuntary ocular oscillations. Alternatively, a malfunction of cerebellar locomotor control might be involved, since DBN is considered a vestibulocerebellar disorder.

**Methods:**

Investigation of walking in 50 DBN patients (age 72±11 years, 23 females) and 50 healthy controls (HS) (age 70±11 years, 23 females) using a pressure sensitive carpet (GAITRite). The patient cohort comprised subjects with only ocular motor signs (DBN) and subjects with an additional limb ataxia (DBNCA). Gait investigation comprised different walking speeds and walking with eyes closed.

**Results:**

In DBN, gait velocity was reduced (p<0.001) with a reduced stride length (p<0.001), increased base of support (p<0.050), and increased double support (p<0.001). Walking with eyes closed led to significant gait changes in both HS and DBN. These changes were more pronounced in DBN patients (p<0.001). Speed-dependency of gait variability revealed significant differences between the subgroups of DBN and DBNCA (p<0.050).

**Conclusions:**

(I) Impaired visual control caused by involuntary ocular oscillations cannot sufficiently explain the gait disorder. (II) The gait of patients with DBN is impaired in a speed dependent manner. (III) Analysis of gait variability allows distinguishing DBN from DBNCA: Patients with pure DBN show a speed dependency of gait variability similar to that of patients with afferent vestibular deficits. In DBNCA, gait variability resembles the pattern found in cerebellar ataxia.

## Introduction

Downbeat nystagmus (DBN) is the most common form of acquired fixation nystagmus [1]. Patients who have DBN typically report dizziness with oscillopsia, especially during eccentric gaze. Within the last decades, the characteristics of these oculomotor abnormalities have been intensively investigated (for overview see [2]). Most patients with DBN also report instability while standing and walking. So far, however, a detailed investigation of their gait behavior is missing. A few studies have focused on the balance control of DBN during stance; the findings of these studies point to increased postural sway in the anterior-posterior direction which intensifies when standing with eyes closed [3].

The majority of DBN patients have cerebellar dysfunction due to toxic, degenerative, inflammatory, or neoplastic pathologies [1]. One third of the patients show an idiopathic form, i.e. no underlying cerebellar disorder can be identified. The pathophysiological model of DBN suggests a dysfunction of vestibulo-cerebellar regions. Brain imaging studies and cerebellar lesion studies give more precise evidence for a hypofunction, a hypometabolism, and a reduction of gray matter volume in the flocculus and paraflocculus [4], [5]. The flocculus and paraflocculus project to the central connections of the anterior semicircular canal and of the otoliths [6] in the ipsilateral superior and medial vestibular nuclei, and the y-group [7]. The relevance of otolith function for DBN is further stressed by its dependence on head position in relation to gravity [8]. The vestibulocerebellum seems to mediate the integration of graviceptive information, thereby stabilizing gaze in the vertical direction [9]. It is not known whether these mechanisms are also relevant for locomotor control.

Two principal processes could contribute to a gait disturbance of DBN patients. First, impaired visual feedback related to oscillopsia during walking might be involved. Previous studies have shown that visual deprivation or perturbation affect multiple aspects of gait kinematics, such as heading direction, walking speed, cadence, stride length, stance phase duration, swing limb trajectory, foot elevation, gait variability, foot positioning and upper body stability [10], [11]. Alternatively, the vestibulocerebellar dysfunction of DBN patients may directly affect locomotor control.

The aim of the current study was to investigate and characterize the gait performance of patients with DBN. We also investigated whether the gait disturbance of DBN patients is mainly affected by impaired visual feedback control during walking or a dysfunction of cerebellar locomotor control.

## Methods

### Ethical standard

The study protocol was approved by the Institutional Review Board of the ethics committee of the Ludwig-Maximilians University Munich (No. 333-07). The study was conducted according to the principles expressed in the Declaration of Helsinki. All subjects gave their informed written consent prior to the experiments.

### Subjects

Fifty patients with downbeat nystagmus (DBN) and 50 age-matched healthy subjects (HS) were recruited in our Dizziness Clinic (German Center for Vertigo and Balance Disorders and Department of Neurology).

Exclusion criteria for DBN patients and HS were concomitant gait disorders due to Parkinson’s disease, stroke, neoplasia of the brain or of the spinal cord, and orthopedic or cardiovascular diseases affecting locomotion. All subjects underwent a complete neurological and physical examination including testing of vestibular, postural, and sensory functions.

### Experimental setup and procedures

Gait analysis was performed using a 6.7-m-long pressure-sensitive carpet (GAITRite, CIR System, Havertown, USA) with a sampling rate of 120 Hz. The carpet system provides the mean values and standard deviations for all relevant gait parameters. The following parameters were analyzed: Functional Ambulation Profile (FAP), a score with linear relationship of step length to leg length ratio to step time when the velocity is “normalized” to leg length [12], velocity, cadence, stride time, stride length, base of support, double support percentage, and the coefficient of variation (CV) of stride time, stride length and base of support as a marker for the magnitude of gait variability. The gait parameters were grouped into gait domains similar to previous studies [13], [14]. Based on a principal factor analysis approach the domains are categorized in the following sections: pace (gait velocity, stride length, cadence), cycle (swing and stance phases), support (base of support, doubles support phases), and variability (CV of stride time and CV of stride length, CV of base of support).

All patients and controls had to walk over the carpet at three different speeds (preferred, slow, and maximally fast). Subsequently, patients were instructed to walk with preferred speed and eyes closed over the carpet. Each condition was tested twice. The spatiotemporal gait parameter values were calculated for each single walk, and the mean of both walks (for each condition) was used for further analysis. After testing of possible side asymmetries (Wilcoxon, Mann-Whitney) for each walking condition, data of both limbs were pooled in order to increase the number of step events. Pooling the data yielded on average 16.3±1.5 steps during walking at preferred speed, 24.2±4.1 steps during slow speed, 14.0±0.5 steps during fast walking, and 17.3±3.2 steps during walking with eyes closed.

### Data analysis

Matlab and SPSS were used for data analysis. A Matlab routine was written to calculate the CV values by using the formula:

CV [%]  =  standard deviation of the parameter *100/mean of the parameter.

Two different statistical models were used: (I) A two-way Analysis of Variance (ANOVA) was performed with the factors “group” (DBN, HS) and “condition” (slow, preferred, maximally fast, eyes closed) and the interaction effect “group×condition”. (II) In order to further determine subgroup differences of the DBN cohort we applied a second, two-way ANOVA model with the factors “subgroup” (pure DBN, DBN plus neuropathy (PNP), DBN plus bilateral vestibular failure (BVF), DBN plus limb ataxia (DBNCA)) and “condition” (slow walking, preferred, maximally fast walking, eyes closed) and the interaction effect “subgroup×condition”. Significant interaction effects were then further decomposed into simple main effects with Bonferroni corrections for both models separately. To quantify the subjects’ ability to walk with eyes closed (EC) compared to walking with eyes open (EO), a Variation Rate (VR) in analogy to the Romberg quotient [15] was calculated with the formula:

VR [%] = 100*(parameter_eyes open_–parameter_eyes closed_)/parameter_eyes open_.

The results were considered significant if p<0.05.

## Results

### Demographic information of the enrolled subjects

Demographic data on DBN patients and HS are given in Table S1. Mean duration of symptoms was 4.2±3.8 years.

The etiology of DBN was mainly idiopathic (∼68%, n = 34). The cohort of DBN with secondary forms comprised patients with sporadic, adult-onset cerebellar atrophy (SAOA) (∼18%, n = 9), alcoholic cerebellar atrophy (∼8%, n = 4), autoimmune cerebellitis (GAD antibody+; ∼4%, n = 2) and midline cerebellar stroke (∼2%, n = 1).

Nine DBN patients (∼18%) also showed limb ataxia which was indicated by a dysmetria during goal-directed limb movements and intentional tremor (seven patients with SAOA, one patient with alcoholic cerebellar atrophy, one patient with GAD+ cerebellitis). Ten DBN patients (∼20%) had a peripheral neuropathy of the legs, which was indicated by a reduction of the ankle jerk reflexes and distal sensory hypofunction (seven patients with idiopathic DBN, two patients with alcoholic cerebellar atrophy, one patient with midline cerebellar stroke). Five DBN patients also had a bilateral vestibular hypofunction with nystagmus <5°/sec after caloric irrigation of the horizontal semicircular canals (30°C/44°C) and pathologic head-impulse tests [16] (four patients with idiopathic DBN, one patient with alcoholic cerebellar atrophy).

One patient had peripheral neuropathy and bilateral vestibular hypofunction (with alcoholic cerebellar atrophy). None of the patients in the DBN cohort had a combination of peripheral sensory loss and cerebellar limb ataxia.

### Gait impairments of DBN patients

**Figure 1:**
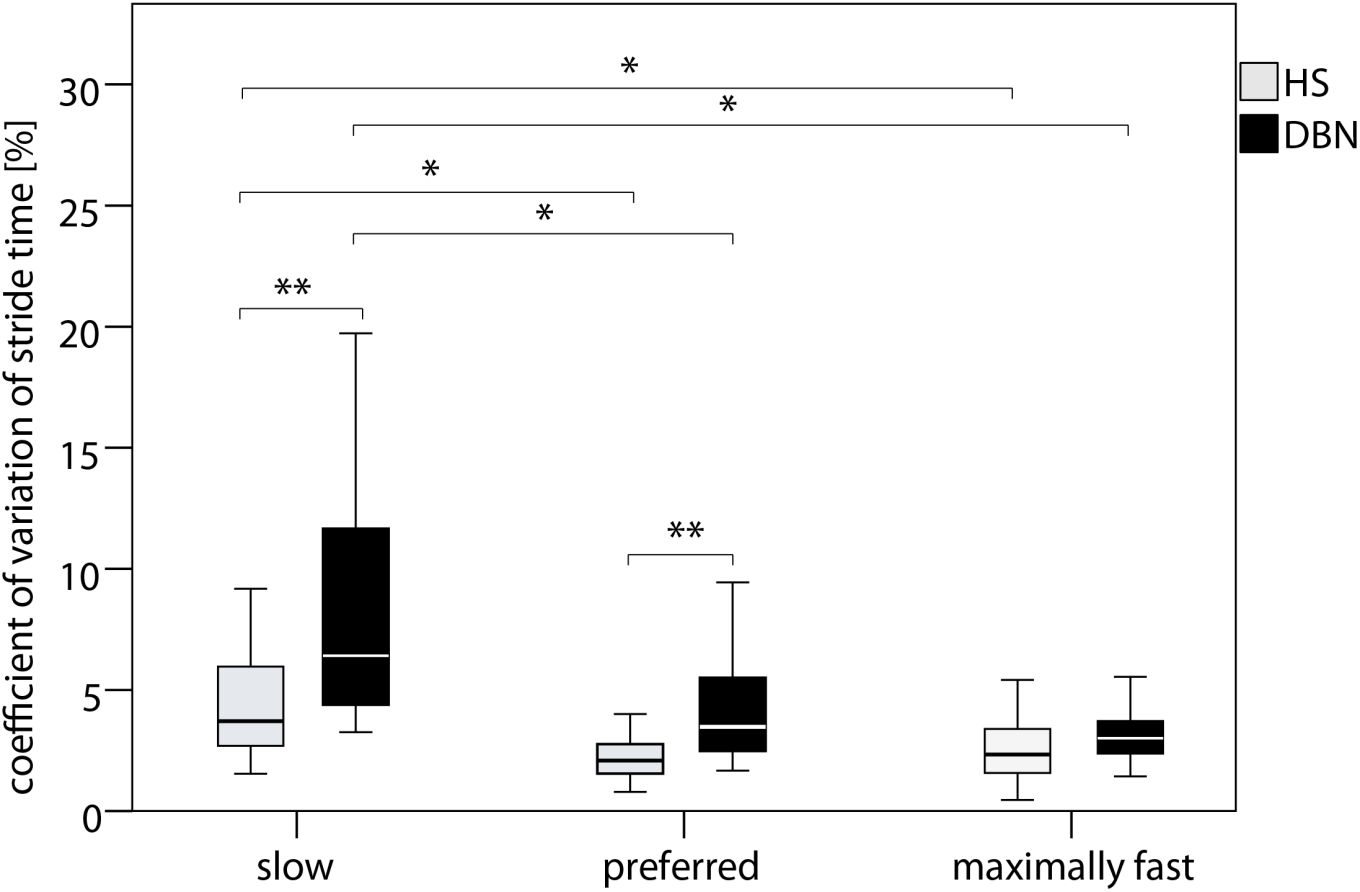
Speed-dependent temporal gait variability. Boxplots with median, sample minimum and maximum, lower and upper quartile for DBN patients (gray) and HS (black) * indicates p<0.05 of the two-way ANOVA (factor “group”: DBN, HS; factor “speed condition” slow, preferred, maximally fast speed) with posthoc Bonferroni posthoc analysis of the interaction effect “group×condition”. Abbreviations: HS - healthy subjects. DBN - downbeat nystagmus syndrome.

The gait performance of DBN patients compared to that of HS was characterized by a significant reduction of gait velocity and stride length (all p<0.001) accompanied by an increase of stride time, base of support, double support percentage, CV of stride length, and CV of stride time (all p<0.001) (Table 1). Significant group×condition effects were found for the parameters gait velocity (p<0.050), stride time (p<0.05) and CV of stride time (p<0.001). Decomposing the significant interaction effect revealed that during “walking with EC” and “walking with preferred speed” the gait velocity was decreased (p<0.050) in DBN patients compared to HS. Stride time (p<0.050) and CV of stride time (p<0.010) were increased in DBN compared to HS during “walking with EC” and “walking with preferred speed”. For “walking with slow speed” we found a significantly increased CV of stride time (p<0.01) in the DBN cohort (Figure 1). For “walking with maximally fast speed” the DBN cohort walked significantly slower than HS (p<0.050).

**Table 1:** Results of the two-way ANOVA model (DBN vs. HS).

Gait domain	Parameter	Group (DBN/HS)	Condition	Group×Condition
Pace	velocity [m/sec]	**F_1_ = 21.71, p<0.001**	**F_3_ = 13.92, p<0.001**	**F_1, 7_ = 3.73, p<0.050**
Pace	cadence [min^−1^]	**F_1_ = 15.92, p<0.001**	**F_3_ = 12.06, p<0.001**	F_1, 7_ = 0.36, n.s.
Pace	stride length [m]	**F_1_ = 27.52, p<0.001**	**F_3_ = 6.02, p<0.010**	F_1, 7_ = 0.61, n.s.
Cycle	stride time [s]	**F_1_ = 18.37, p<0.001**	**F_3_ = 9.34, p<0.001**	**F_1, 7_ = 2.47, p<0.050**
Cycle	double support percentage [%]	**F_1_ = 15.32, p<0.001**	**F_3_ = 28.83, p<0.001**	F_1, 7_ = 1.91, n.s.
Support	base of support [m]	**F_1_ = 7.81, p<0.010**	**F_3_ = 4.86, p<0.010**	F_1, 7_ = 0.26, n.s.
Variability	CV of stride time [%]	**F_1_ = 11.79, p<0.001**	**F_3_ = 15.79, p<0.001**	**F_1, 7_ = 6.05, p<0.001**
Variability	CV of stride length [%]	**F_1_ = 17.08, p<0.001**	**F_3_ = 30.6, p<0.001**	F_1, 7_ = 1.50, n.s.
Variability	CV of base of support [%]	F**_1_** = 0.296, n.s.	F_3_ = 0.27, n.s.	F_1, 7_ = 1.28, n.s.

F-Values and p-values are indicated for a 2-way ANOVA (factor “group”: HS, DBN; factor “condition”: walking with slow, preferred and maximal speed, walking with eyes closed).

Abbreviations: HS - healthy subjects. DBN - downbeat nystagmus syndrome. CV - coefficient of variation.

**Figure 2:**
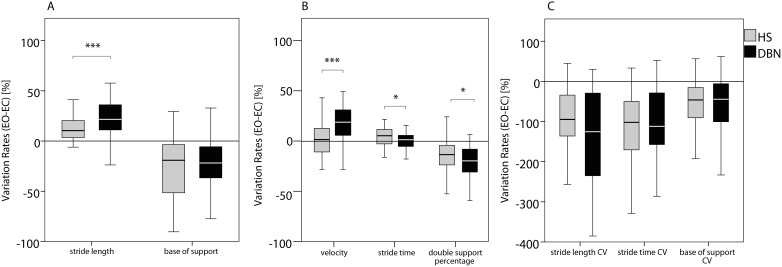
Effect of absent visual control on gait variables in DBN and HS. Box plots of the individual Variation Rates with median, sample minimum and maximum, lower and upper quartile for healthy subjects (grey) and patients with DBN (black). Gait variables were grouped in spatial (A), temporal (B), and variability (C) gait parameters. Conventional spatiotemporal gait parameters were worse in patients with DBN under walking with closed eyes, indicating a higher stabilizing effect of vision on balance control, despite the ocular oscillations in DBN. Note the scale enlargement in the section C of the figure. * indicates p<0.050, ** indicates p<0.010, *** indicates p<0.001 of the two-way ANOVA (factor “group”: HS, DBN; factor “condition”: eyes opened, eyes closed) with Bonferroni posthoc analysis of the interaction effect “group×condition” Abbreviations: HS - healthy subjects. DBN - downbeat nystagmus syndrome. EO - eyes opened. EC - eyes closed.

Individual differences between walking with EO compared to EC, calculated as Variation Rates (Table 2), were higher in DBN than in HS for the parameters FAP, gait velocity, stride length (all p<0.001), stride time (p<0.050), and double support percentage (p<0.050) (Figure 2). Variation Rates for the CV of stride length, CV of stride time and CV of base of support showed a high data variance in both groups without any significant group differences.

**Table 2:** Relative gait changes during walking with eyes opened and eyes closed.

	HS	DBN	F	p-value
Walking with eyes closed				
VR for FAP [%]	6.9±10.4	18.4±18.3	14.675	**<0.001**
VR for gait velocity [%]	6.7±19.0	23.1±18.2	18.957	**<0.001**
VR for cadence [%]	1.6±11.8	−0.6±10.8	1.315	n.s.
VR for stride length [%]	12.5±11.5	23.2±16.6	13.753	**<0.001**
VR for base of support [%]	−26.1±19.7	−20.5±17.1	0.523	n.s.
VR for stride time [%]	4.7±9.8	−0.6±10.9	6.715	**<0.050**
VR for double support percentage [%]	−11.0±17.4	−18.8±20.4	4.232	**<0.050**
VR for stride length CV [%]	−**142.1±133.8**	−**115.7±118.6**	1.067	n.s.
VR for base of support CV [%]	−**62.6±85.0**	−**51.0±74.3**	1.911	n.s.
VR for stride time CV [%]	−**109.1±131.4**	−**131.7±122.4**	0.779	n.s.

Mean values and standard deviation of the Variation Rates for the different gait variables under walking with eyes closed in respect to walking with eyes opened. F-Values and p-values are indicated for a one-way ANOVA (factor “group”: HS, DBN).

Abbreviations: HS - healthy subjects. DBN - downbeat nystagmus syndrome. VR - Variation Rates.

Pearson’s correlations between the duration of symptoms of DBN and the gait parameters revealed significant negative coefficients for FAP, velocity, stride length, swing percentage, and significant positive coefficients for double support percentage, stance percentage and the CV of stride length, all under the condition of “walking with EC” (Table S2). For other gait conditions (e.g., EO conditions), no significant correlation between the duration of symptoms and gait parameters were found.

We also found significant correlations between the Variation Rates (EO – EC) and the duration of symptoms for the parameters gait velocity (Figure S1), FAP, cadence, stride length, stride time, double support percentage, swing percentage, stance percentage, the CV of base of support, and the CV of stride length (Table S2).

### Gait performance and concomitant symptoms

**Figure 3:**
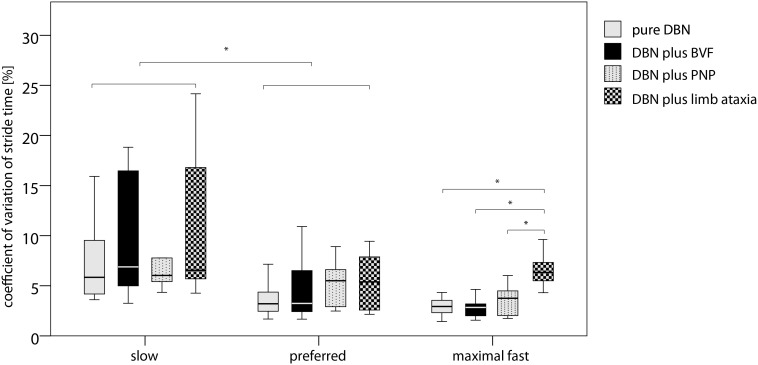
Speed-dependent temporal gait variability of the DBN subgroups. Boxplots with median, sample minimum and maximum, lower and upper quartile for patients with pure DBN (gray), patients with DBN plus BVF (black), patients with DBN plus PNP (gray with black dots) and patients with DBN plus limb ataxia (gray with black squares). * indicates p<0.050, ** indicates p<0.010, *** indicates p<0.001 of the two-way ANOVA (factor “group”: pure DBN, DBN plus BVF, DBN plus PNP, DBNCA; factor “condition”: slow, preferred, maximally fast speed) with Bonferroni posthoc analysis of the interaction effect “group×condition” Abbreviations: DBN - downbeat nystagmus syndrome. BVF - bilateral vestibular failure. PNP - peripheral sensory neuropathy.

Subgroup analysis for the DBN cohort showed significant group effects for the parameters cadence, stride length, stride time, double support percentage, CV of stride time, CV of stride length (all p<0.001), and base of support (p<0.010). Significant subgroup×condition effects were found for stride time (p<0.050) and CV of stride time (p<0.001) (Table 3). Decomposing the significant interaction effects revealed that the stride time was higher in DBNCA compared to all other subgroups (p<0.050) during all examined walking conditions. The CV of stride time was significantly higher in DBNCA compared to all other DBN subgroups for the condition “walking with maximally fast speed” (p<0.010) (Figure 3).

**Table 3:** Results of the two-way ANOVA model (DBN subgroups).

Gait domain	Parameter	Group (DBN/DBN+BVF/DBN+PNP/DBNCA)	Condition	Group×Condition
Pace	velocity [m/sec]	F_3_ = 1.25, n.s.	**F_3_ = 13.92,** **p<0.001**	F_3, 15_ = 1.73, n.s.
Pace	cadence [min^−1^]	**F_3_ = 15.92, p<0.001**	**F_3_ = 12.06,** **p<0.001**	F_2, 15_ = 0.36, n.s.
Pace	stride length [m]	**F_3_ = 27.52, p<0.001**	**F_3_ = 6.02,** **p<0.010**	F_3, 15_ = 0.61, n.s.
Cycle	stride time [s]	**F_3_ = 18.37, p<0.001**	**F_3_ = 9.34,** **p<0.001**	**F_3, 15_ = 2.47, p<0.050**
Cycle	double support percentage [%]	**F_3_ = 15.32, p<0.001**	**F_3_ = 28.83,** **p<0.001**	F_3, 15_ = 1.91, n.s.
Support	base ofsupport [m]	**F_3_ = 7.81, p<0.010**	**F_3_ = 4.86,** **p<0.010**	F_3, 15_ = 0.26, n.s.
Variability	CV of stridetime [%]	**F_3_ = 11.79, p<0.001**	**F_3_ = 15.79,** **p<0.001**	**F_3, 15_ = 6.05, p<0.001**
Variability	CV of stridelength [%]	**F_3_ = 17.08, p<0.001**	**F_3_ = 30.6,** **p<0.001**	F_3, 15_ = 1.50, n.s.
Variability	CV of base ofsupport [%]	F**_3_** = 0.296, n.s.	F_3_ = 0.27, n.s.	F_3, 15_ = 1.28, n.s.

F-Values and p-values are indicated for a 2-way ANOVA (factor “group”: DBN/DBN+BVF/DBN+PNP/DBNCA; factor “condition”: walking with slow, preferred and maximal speed, walking with eyes closed).

Abbreviations: HS - healthy subjects. DBN - downbeat nystagmus syndrome. BVF - bilateral vestibular failure. PNP - sensory neuropathy. CA - cerebellar ataxia. CV - coefficient of variation.

## Discussion

Our main findings are as follows:

1. Patients with DBN show gait impairments which depend on the current walking condition and on the concomitant symptoms of the patients.
2. Gait impairments of DBN patients depend on the actual walking speed: slow walking is preferentially affected.
3. Impaired visual control caused by involuntary ocular oscillations cannot sufficiently explain the DBN gait disorder.

### Gait characteristics of patients with DBN

The gait of DBN patients was characterized by changes of temporal and spatial variables of the pace, cycle, support, and variability domains. Reduction of the walking pace was an overall feature of DBN gait in nearly all gait conditions, and appears to be in conflict with previous findings in patients with classical cerebellar deficits [17], [18]. The current concept of cerebellar pacemaker function in the locomotor network is based on animal findings [19] and human findings [20] of a midline cerebellar region controlling for the stepping frequency and pace. We have recently found that walking speeds and stepping frequency of patients with cerebellar deficits were mainly reduced during fast walking, but normal at preferred walking [21], [22]. These findings underline the hypothesis that cerebellar pacemaker function is maximally loaded for high walking speeds. In this line, the overall reduction of walking pace of DBN patients (under slow, preferred, and maximally fast walking speeds) could also reflect a change of the dynamic stability strategy; the patients reduce their walking speeds and change their gait cycles, e.g., they reduce swing phases and consecutively increase stance and double support phases. Together with an increased base of support, these changes have a stabilizing effect on the dynamic balance control during walking [18].

Gait variability, which describes the stride-to-stride fluctuations, is known to be a robust marker for gait stability. Thus, gait variability reflects rather gait pathology than compensatory gait impairments. It is known to be related to the risk of falling in patients [23], [24]. DBN patients showed increased levels of gait variability in the fore-aft, but not in the medio-lateral direction. This is in line with previous findings of patients with other cerebellar disorders, who have increased levels of temporal and spatial gait variability [21], [22]. The further implications of this finding will be discussed in the section entitled *“Speed-dependency of gait in DBN”*.

### Gait of DBN patients with eyes closed

Visual feedback is used in HS to control dynamic balance during gait, to control path integration, foot trajectory and placement, and the adaptation of gait [11], [25]. For DBN patients, however, visual information is less reliable. Oscillopsia due to the involuntary eye movements substantially impairs their ability to fixate targets. The deterioration of visual control might affect the gait performance of DBN patients. If impaired visual control is the main contributor to gait impairment, one would expect a reduced dependence of gait dynamics on the visual system, which would be expressed in a stable or even improved walking behavior when the disturbing visual input is absent (EC condition). However, our results contradict this hypothesis since gait variables of DBN patients deteriorated during absent visual control. This indicates that the disturbed locomotor control of DBN patients cannot be exclusively explained by a disturbed visual control due to oscillopsia. Alternative pathophysiological hypotheses include the direct coupling of the oculomotor system and the locomotor system (thus independent on visual scene perception and retinal slip). Previous investigations of the influence of eye movements per se on postural control support this view [26], [27].

Alternatively, there could be a separate, direct functional connection from the vestibulocerebellum to the postural and locomotor network (further discussion in the next section).

Individual Variation Rates between EO and EC were higher in DBN patients than in HS, indicating that DBN patients utilize visual inputs during walking even more. This phenomenon was independent from concomitant sensory deficits in DBN patients and reflects a second feature of DBN gait control. The weighting of sensory inputs of DBN patients seems to be shifted towards the visual system, a mechanism not expected a priori in a disorder characterized by nystagmus and oscillopsia. Such a shift in sensory dependency during stance and gait can be attributed to compensatory postural strategies, supported by the finding of significant correlations between the duration of symptoms of DBN and the functional decline of gait parameters when the visual feedback is absent. In this sense, we suggest that DBN patients develop a vision-based compensatory strategy over a longer term. For stance control of single subjects with DBN, it was likewise found that patients utilize visual information for the control of postural sway [3].

### Speed-dependency of gait in DBN

There are several lines of evidence for a speed-dependent integration of sensory information into the locomotor network. Dogs and humans with acute unilateral vestibulopathy are better off running than walking [28]. Using motor imagery in fMRI, activations of sensory cortex areas were shown to decrease and midline cerebellar activity increase during fast locomotion [29]. The role of the cerebellum in speed control is also supported by animal experiments showing that fast locomotion is achieved by highly automated central pattern generators in the spinal cord, which are mainly driven by cerebellar pacemakers [30], [31]. On a behavioral level, investigations of speed-dependent gait variability revealed characteristic patterns for patients with bilateral vestibular failure and with cerebellar ataxia [21], [22]. Both patient groups have increased levels of gait variability during slow locomotion and minimal, near-to-normal, values of gait variability during preferred walking. During fast walking, gait variability of bilateral vestibular failure further decreases while gait variability of cerebellar ataxia patients increases again.

In this study both, HS and DBN patients showed a speed-dependence of gait variables, but this speed-dependence appears to be different for the two groups.

DBN patients exhibited increased temporal gait variability at slow and preferred walking speeds, but not at fast walking speeds, thus showing a speed-dependency of gait variability similar to that of patients with bilateral vestibular failure [21]. In patients with bilateral vestibular failure this was interpreted to reflect a disturbed vestibular feedback control during slow locomotion, when sensory feedback mechanisms are maximally loaded. This pattern was also present in the DBN subgroup without an afferent sensory deficit, so that a central origin of this speed-dependence can be assumed.

A possible, but not proven, explanation could be that the dysfunction of the vestibulocerebellum in DBN patients leads to an insufficient sensory integration into locomotion control. This could result in speed-dependent gait changes comparable to those of patients with afferent sensory deficits. The function of the vestibulocerebellum has already been intensively investigated in the context of the ocular motor system. Here, the flocculus is known to integrate vestibular signals [32] for the adaptation of vertical smooth pursuit and gaze holding. Moreover, ocular motor deficits of DBN patients have been shown to depend on gravity (and thereby on vestibular signals) and a model of a disturbed otolith-ocular interaction in DBN patients was established [33], [34].

Gait variability analysis during fast walking revealed two different groups of patients with DBN. Patients who also have symptoms of a cerebellar hemisphere involvement (indicated by the presence of limb ataxia) showed high temporal gait variability during fast walking. This pattern is comparable to one that we recently found for patients with global cerebellar ataxia [21]. DBN patients without limb ataxia were found to have normal temporal gait variability at walking with maximally fast speed. Thus, vestibulocerebellar control of locomotion differs from (hemispheric) cerebellar control in terms of a sufficiently functioning cerebellar pacemaker during fast walking.

## Conclusion

Impaired locomotion in DBN patients is not solely caused by visual disturbances associated with involuntary eye movements. Instead, it reflects a deficit which may be explained by impaired central vestibular integration. Alternatively, direct connections between the oculomotor system and the locomotor system might be present.

## Supporting Information

Figure S1 **Correlation of the duration of symptoms and the effect of visual control on gait speed.** Pearson’s correlation of the 50 individuals with DBN (black dots). The black lines indicate the correlation coefficient (inner line) with 0.95 confidential interval (outer lines). Abbreviations: EO - eyes open. EC - eyes closed.
(TIF)

Table S1 **Demographic, clinical and paraclinical characteristics of the enrolled subjects.** Abbreviations: HS - healthy subjects. DBN - downbeat nystagmus syndrome. GAD - glutamate-decarboxylase. MRI - magnetic resonance imaging.
(DOCX)

Table S2 **Correlations between the duration of symptoms, the gait parameters and the Pearson’s correlations with coefficient (R) and p-values between the duration of symptoms [months] and the Variation Rates for the different gait parameters.** Abbreviations: HS - healthy subjects. DBN - downbeat nystagmus syndrome. CV - coefficient of variation. VR - Variation Rates.
(DOCX)
