# Supplementary figures and images for: The Gait Disorder in Downbeat Nystagmus Syndrome
Source: PLoS One. 2014 Aug 20;9(8):e105463. doi: 10.1371/journal.pone.0105463 (PMC4139349; doi:10.1371/journal.pone.0105463)

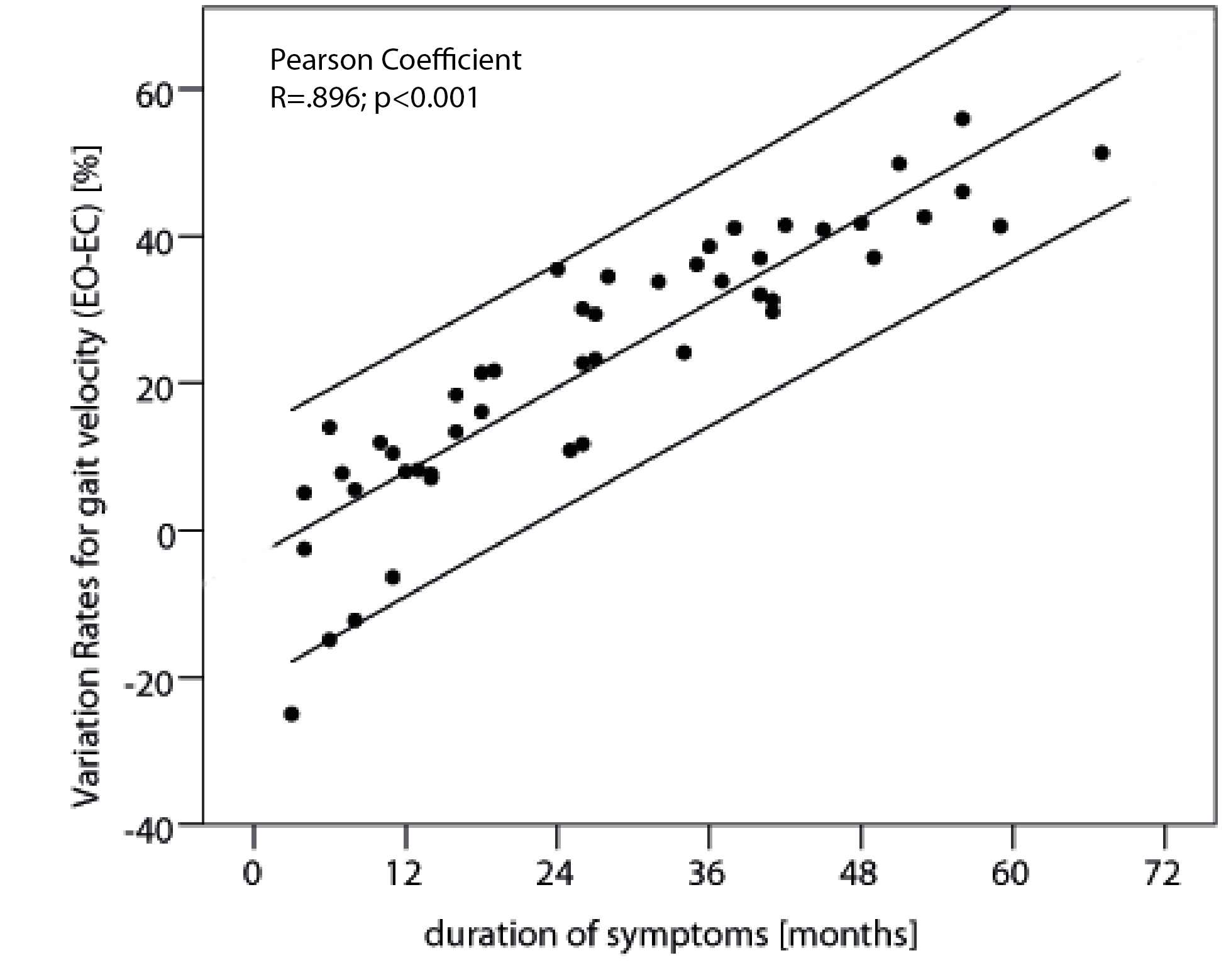

Supplement: Figure S1 — Correlation of the duration of symptoms and the effect of visual control on gait speed. Pearson’s correlation of the 50 individuals with DBN (black dots). The black lines indicate the correlation coefficient (inner line) with 0.95 confidential interval (outer lines). Abbreviations: EO - eyes open. EC - eyes closed. (TIF) [file pone.0105463.s001.tif]
