# Supplementary material for: The Gait Disorder in Downbeat Nystagmus Syndrome
Source: PLoS One. 2014 Aug 20;9(8):e105463. doi: 10.1371/journal.pone.0105463 (PMC4139349; doi:10.1371/journal.pone.0105463)
Supplement: Table S1 — Demographic, clinical and paraclinical characteristics of the enrolled subjects. Abbreviations: HS - healthy subjects. DBN - downbeat nystagmus syndrome. GAD - glutamate-decarboxylase. MRI - magnetic resonance imaging. (DOCX) [file pone.0105463.s002.docx]

Table S4

|  | **HS** | **DBN** |
| --- | --- | --- |
| **Basic demographic information** |  |  |
| gender female : male | 23 : 27 | 23 : 27 |
| mean age in years | 72 ± 11 | 70 ± 11 |
| mean leg length in meters | 0.90 ± 0.11 | 0.89 ± 0.09 |
| mean height in meters | 1.78 ± 0.13 | 1.76 ± 0.12 |
| mean duration of symptoms in years | - | 4.2 ± 3.8 |
| **Etiology of DBN** |  |  |
| idiopathic | - | 34 |
| sporadic adult onset ataxia |  | 9 |
| others |  | 7 |
| *GAD+ cerebellitis*  *ethyltoxic atrophy*  *midline cerebellar stroke* |  | *2*  *4*  *1* |
| **Concomitant symptoms** |  |  |
| no symptoms | 50 | 26 |
| sensory neuropathy of the legs | - | 10 |
| bilateral vestibular hypofunction | - | 5 |
| cerebellar limb ataxia | - | 9 |
| **Oculomotor findings** |  |  |
|  |  |  |
|  |  |  |
| **Neuroimaging findings** |  |  |
| MRI performed | 0 | 30 |
| Cerebellar atrophy | - | 9 |
| Cerebellar WML | - | 12 |
| Cerebellar postischemic lesions | - | 1 |
| supratentorial atrophy | - | 4 |
| supratentorial WML | - | 19 |
| supratentorial postischemic lesions | - | 3 |

Legend: demographic and clinical characteristics of the enrolled subjects

Abbreviations: HS - healthy subjects DBN - downbeat nystagmus syndrome GAD - glutamate-decarboxylase WML - white matter lesions
