# Supplementary material for: The Gait Disorder in Downbeat Nystagmus Syndrome
Source: PLoS One. 2014 Aug 20;9(8):e105463. doi: 10.1371/journal.pone.0105463 (PMC4139349; doi:10.1371/journal.pone.0105463)
Supplement: Table S2 — Correlations between the duration of symptoms, the gait parameters and the Pearson’s correlations with coefficient (R) and p-values between the duration of symptoms [months] and the Variation Rates for the different gait parameters. Abbreviations: HS - healthy subjects. DBN - downbeat nystagmus syndrome. CV - coefficient of variation. VR - Variation Rates. (DOCX) [file pone.0105463.s003.docx]

Table S5

|  | Duration of symptoms [months] | |
| --- | --- | --- |
| **Walking with eyes closed** | coefficient | p-value |
| FAP | **-0.501** | **<0.001** |
| gait velocity | **-0.479** | **<0.001** |
| cadence | -0.105 | n.s. |
| stride length | **-0.497** | **<0.001** |
| base of support | 0.014 | n.s. |
| stride time | 0.139 | n.s. |
| double support percentage | **0.344** | **<0.050** |
| Mean stride length CV | **0.462** | **<0.001** |
| Mean base of support CV | -0.222 | n.s. |
| Mean stride time CV | 0.175 | n.s. |
| VR for FAP | **0.412** | **<0.010** |
| VR for gait velocity | **0.896** | **<0.001** |
| VR for cadence | **0.428** | **<0.010** |
| VR for stride length | **0.787** | **<0.001** |
| VR for base of support | 0.075 | n.s. |
| VR for stride time | **-0.450** | **<0.001** |
| VR for double support percentage | **-0.661** | **<0.001** |
| VR for stride length CV | **-0.456** | **<0.010** |
| VR for base of support CV | 0.360 | n.s. |
| VR for stride time CV | -0.278 | n.s. |
